# Time Course of Sensory Substitution for Gravity Sensing in Visual Vertical Orientation Perception following Complete Vestibular Loss

**DOI:** 10.1523/ENEURO.0021-20.2020

**Published:** 2020-07-07

**Authors:** Dora E. Angelaki, Jean Laurens

**Affiliations:** 1 Center for Neural Science and Tandon School of Engineering, New York University, NY 10003,; 2 Department of Neuroscience, Baylor College of Medicine, Houston, TX 77030

## Abstract

Loss of vestibular function causes severe acute symptoms of dizziness and disorientation, yet the brain can adapt and regain near to normal locomotor and orientation function through sensory substitution. Animal studies quantifying functional recovery have yet been limited to reflexive eye movements. Here, we studied the interplay between vestibular and proprioceptive graviception in macaque monkeys trained in an earth-vertical visual orientation (subjective visual vertical; SVV) task and measured the time course of sensory substitution for gravity perception following complete bilateral vestibular loss (BVL). Graviceptive gain, defined as the ratio of perceived versus actual tilt angle, decreased to 20% immediately following labyrinthectomy, and recovered to nearly prelesion levels with a time constant of approximately three weeks of postsurgery testing. We conclude that proprioception accounts for up to 20% of gravity sensing in normal animals, and is re-weighted to substitute completely perceptual graviception after vestibular loss. We show that these results can be accounted for by an optimal sensory fusion model.

## Visual Abstract

**Figure:**
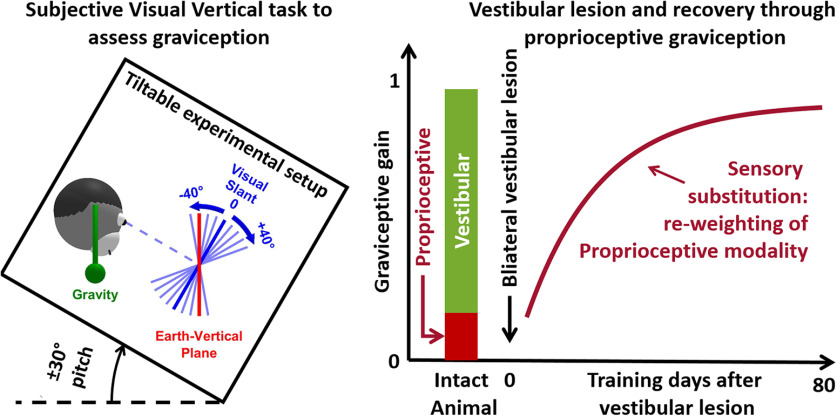

## Significance Statement

Verticality perception is based on gravity sensing by the vestibular organs in the inner ear and by trunk proprioceptors. Here, we measured the contribution of vestibular and proprioceptive signals to verticality perception in macaque monkeys before and following complete vestibular lesion. We found that proprioception contributes to 20% of gravity sensing in healthy animals. Following vestibular loss, gravity sensing recovers to baseline levels in weeks and is affected by how often the animals performed the task. Comparison with previous experimental and modeling studies indicate that our results likely generalize to gravity sensing in humans, at least at small (<30°) tilt angles.

## Introduction

The vestibular organs in the inner ear are fundamental for several brain functions, including locomotion, gaze stabilization, and spatial navigation. Loss of the vestibular organs in animal models (Thomson et al., 1991; Macpherson and Inglis, 1993) or humans (Crawford, 1964) is immediately followed by a complete inability to stand and severe disorientation. These symptoms improve over a few days and, in a chronic state, patients with bilateral vestibular loss (BVL) can typically perform everyday tasks such as walking or driving but retain equilibrium issues, gaze instability (Gillespie and Minor, 1999; Zingler et al., 2008; Ward et al., 2013; Strupp et al., 2016) as well as cognitive (Hanes and McCollum, 2006; Bigelow and Agrawal, 2015; Popp et al., 2017) and navigation (Schautzer et al., 2003; Brandt et al., 2005) deficits.

Recovery from BVL appears to be based on sensory substitution. Following BVL, gaze stabilization reflexes that counterrotate the eye to compensate for head rotation rely increasingly on visual signals (Huygen et al., 1989) and neck proprioception (Huygen et al., 1991; Sadeghi et al., 2012). Accordingly, neurophysiological studies have shown that vestibular nuclei neurons develop an increased sensitivity to proprioceptive stimulation (Yates and Miller, 2009; Sadeghi et al., 2012; McCall et al., 2013) and efference copies (Sadeghi et al., 2012) following vestibular lesions.

Gravity plays a critical role in shaping our experience of the world, influencing both sensory perception and motor planning (Indovina et al., 2005; Zago and Lacquaniti, 2005; MacNeilage et al., 2007; Gaveau et al., 2011; Senot et al., 2012). Gravity sensing arises mainly from the vestibular system, and is a major component of postural control (Mergner et al., 2003), gaze stabilization (Merfeld, 1995; Laurens et al., 2011), central processing of self-motion information (Laurens and Angelaki, 2011, 2017; Laurens et al., 2013), as well as spatial cognition, such as three-dimensional spatial orientation (Oman, 2007; Jeffery et al., 2013; Laurens et al., 2016; Angelaki et al., 2020).

Furthermore, the visual scene and objects around us generally remain perceptually invariant relative to the allocentric world, regardless of our head/body orientation (Van Beuzekom and Van Gisbergen, 2000; Van Beuzekom et al., 2001; De Vrijer et al., 2008, 2009). Clinical studies show that deficits in the vestibular system or in combining gravitational and visual signals due to brain injury compromise this visual stability (Brandt et al., 1994; Funk et al., 2010, 2011; Baier et al., 2012a,b; Guardia et al., 2012). Along with other symptoms, this may result in disturbing episodes in which the world appears upside-down or sideways. For this reason, earth-vertical visual orientation [often referred to as subjective visual vertical (SVV)] tasks, that probe the ability to discern the orientation of visual stimuli relative to gravitational vertical, are a common technique to investigate vestibular function (Tarnutzer et al., 2009a,b; Clemens et al., 2011; Alberts et al., 2015, 2016, 2018), notably in clinical settings (Böhmer and Rickenmann, 1995; Valko et al., 2011). In these tasks, a subject is rolled ear-down in the dark and asked to orient a dimly lit bar vertically in space (to align it with gravity), or to report whether a visual bar is oriented left or right relative to vertical. With only the bar visible, this is accomplished with relatively small errors when the subject is rolled up to ∼45° (Mittelstaedt, 1983; Van Beuzekom and Van Gisbergen, 2000; Kaptein and Van Gisbergen, 2004; De Vrijer et al., 2008). Results from electrical stimulation of vestibular afferents (Lewis et al., 2013) further point to a dominant role of vestibular over proprioceptive systems in creating a gravity-centered visual representation. Although these tasks are mainly used to probe orientation perception in humans, some studies (Daddaoua et al., 2008; Lewis et al., 2008) succeeded in training macaque monkeys to report SVV.

Strikingly, several studies (Tabak et al., 1997; Lopez et al., 2007; Grabherr et al., 2011; Alberts et al., 2015, 2018; Toupet et al., 2017; Bürgin et al., 2018) have shown that average SVV is unaltered in patients suffering from BVL, although these settings may become more variable (Bürgin et al., 2018). This suggests either that the vestibular system contributes little to SVV or that extravestibular gravity sensing can thoroughly substitute for otolith function following vestibular loss. Indeed, a number of studies have shown that graviception naturally occurs in the trunk (Mittelstaedt, 1992, 1996; Vaitl et al., 2002; Trousselard et al., 2003; Clemens et al., 2011; Alberts et al., 2016). Model-based analyses (Clemens et al., 2011; Alberts et al., 2016) have postulated that the brain fuses vestibular and body graviception according to their respective reliabilities, the former typically being more accurate and consequently more heavily weighted. It has been proposed that sensory substitution involves a re-weighting of the remaining sensory modalities (Alberts et al., 2018). However, the normal weight of vestibular cues has never been measured directly, and the time course of sensory reweighting after vestibular loss remains unknown since SVV studies in human patients have always been performed in chronic stages of BVL.

Here, we used an animal model to measure earth-vertical visual orientation perception following vestibular loss. We first tested animals before, and acutely after vestibular loss to test the hypothesis that vestibular cues normally dominate graviception; under this hypothesis, task performance should drop substantially after vestibular loss. Second, we monitored sensory reweighting over time, testing the hypothesis of full recovery and, for the first time, measuring the time course of this recovery. We find a vestibular dominance in graviception, and a progressive restoration of perceptual performance after BVL, with a time constant of approximately three weeks of retraining.

## Materials and Methods

### Animals

Three male rhesus macaques (animals N, P, and Z; aged seven, three, and six years, respectively), were used in the study. Animals were pair-housed in a vivarium under normal day/night cycle illumination. Animals were chronically implanted with a circular delrin ring for head restraint (Gu et al., 2006) and scleral search coils for monitoring eye position. Because of eye coil failures in animal N, an infrared optical eye tracker (ISCAN) monitored binocular eye movements in some experiments. Animals learned to perform a visual orientation discrimination task through standard operant conditioning procedures. After reaching baseline levels, animals N and Z received bilateral labyrinthectomies by opening the vestibular labyrinth, removing the neuroepithelia, and filling the vestibule with streptomycin powder (2%; Newlands et al., 2001; Liu et al., 2013; Yu et al., 2014). We chose to use animals that were previously trained at performing a slant discrimination task (Elmore et al., 2019) so as to minimize the number of animals used in our laboratory. All surgical procedures were conducted under isoflurane anesthesia. Experimental procedures were in accordance with National Institutes of Health guidelines and approved by a local Animal Studies Committee.

### Setup and stimuli

Animals sat in a primate chair 30 cm from an LCD monitor mounted at eye level. Visual stimuli, generated with OpenGL, appeared through a circular aperture subtending ∼29° of visual angle, which was constructed from black, nonreflective cardboard. The same material enclosed the area surrounding the animal’s head and the monitor, such that only the stimulus was visible to the animal. Stimuli were slanted planar surfaces depicting static random dot stereograms (RDSs) and rendered as three-dimensional red-green anaglyphs that filled the viewable region of the monitor and had uniform density on the screen (Sanada et al., 2012; Elmore et al., 2019). We used RDS planes to preclude the use of monocular cues for judging slant so that the task required three-dimensional visual perception. Both the chair and the monitor were attached to a three-dimensional rotating platform (Acutronics Inc.), which pitched the animal and monitor at discrete angles about the interaural axis. Custom Spike2 scripts controlled the trial structure (CED).

### Earth-vertical visual orientation discrimination task

**Figure 1.**
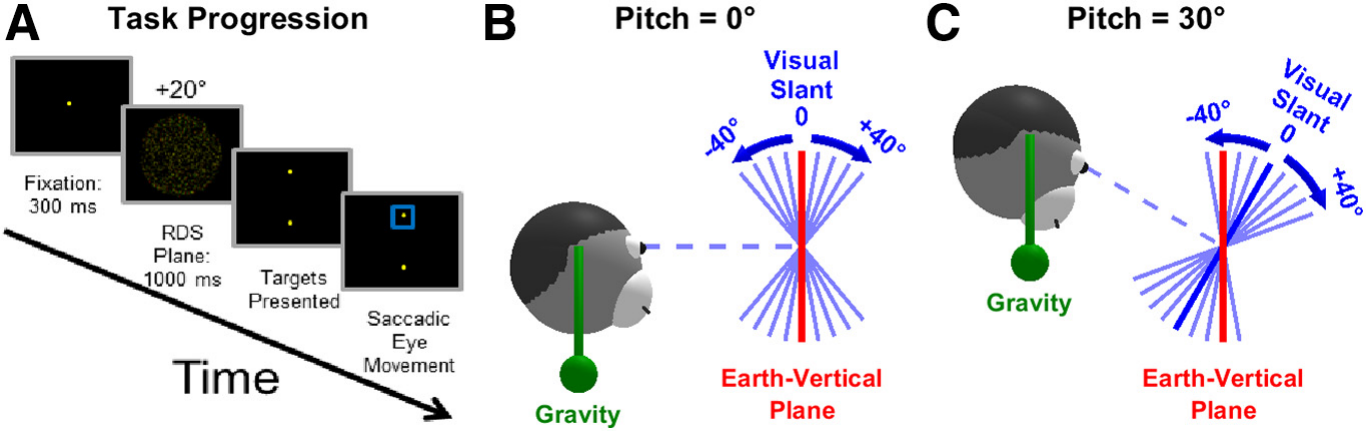
Overview of the task. ***A***, Task flow diagram. Animals must fixate a central point for 300 ms, after which a RDS with a visual slant of ±40° (in intervals of 5°) is shown for 1000 ms. Animals must next report the allocentric orientation of the stereogram by performing an upward or downward saccade. ***B***, ***C***, Coordinate transformations involved in the task. The animals must use gravity sensing (symbolized by a green pendulum) to determine the orientation of the slanted visual stimulus (blue) relative to the allocentric earth-vertical plane (red). If animals perform the task perfectly, then the PSE occurs when the visual stimulus is aligned with the earth-vertical plane, i.e., at 0° slant in ***B*** and −30° slant in ***C***.

We trained animals to perform a SVV task adapted to rotation in the pitch plane. The layout of the task is shown in Figure 1. Each trial began with the presentation of a fixation target at eye level, centered in the viewable region of the LCD screen. Fixation was enforced with a 2° monocular window and 1° vergence window. After fixation for 300 ms, a RDS plane appeared for 800–1000 ms (Fig. 1*A*), and the animal had to maintain fixation during this period (otherwise the trial was aborted). Following stimulus presentation, the fixation point and RDS plane disappeared, and two choice targets appeared 8.6° above and below the center of the screen. Monkeys were trained (see below) to discriminate the orientation of the RDS stimulus relative to an allocentric earth-vertical orientation, defined as the plane whose normal is perpendicular to the direction of gravity. To receive a juice reward, the animal made a saccade to the upper target if he perceived the top of the presented RDS plane to be behind the earth-vertical plane and to the lower target if he perceived the top of the presented RDS plane to be in front of the allocentric earth-vertical plane. Correct answers (rewarded with a drop of water or juice) depend on the magnitude of the monkey’s pitch, such that an accurate percept of one’s body orientation is required for accurate task performance.

For each block of trials on a given day, the monkey was positioned at three different body pitches. A 0° (upright) body pitch was always included, as well as symmetrical positive and negative pitches (e.g., ±30°). The orientation of the RDS planes with respect to the monitor ranged from −40° to 40° in 5° increments, and all planar slant values are presented once in random order at one pitch angle before the monkey’s pitch was changed. The need to use large (5°) increments was dictated by two factors. (1) The range of visual slant stimuli presented should always be the same and independent of the monkey pitch angle. Given that the largest body tilt we used was ± 30°, the visual slant stimuli needed to span a ± 40° range (Fig. 1*B*). (2) The number of trials should be tractable, so the smallest possible difference in visual stimulus slant was set to 5°. This coarse sampling of slant orientations did not allow accurate threshold measurements.

Each pitch angle was repeated between 5 and 33 times (mean: 20.2; SD: 6.8) within each session. Importantly, since the monitor, animal and surrounding enclosure rotated together, the stimulus set was identical in a head-centered reference frame for every pitch angle. In the absence of visual verticality cues, the animal had to rely on other senses to judge the direction of gravity (Rosenberg and Angelaki, 2014). Furthermore, since the animals were held at each static tilt position for a long duration (while all slant angles were tested), a contribution of the semi-circular canals was negligible.

### Behavioral training

All animals were first trained in a slant discrimination task while sitting upright (animal pitch = 0°; Elmore et al., 2019). Following neural recordings, animals were trained with the allocentric vertical task, initially with pitch magnitudes of ±30° and 0°. A reference plane (similar to the reference line of Daddaoua et al., 2008) aligned with the allocentric vertical plane was flashed for 300 ms before stimulus presentation to aid the animal’s decision. The contrast of this reference plane was then gradually reduced. Once animals became proficient to perform the allocentric vertical task at ±30° pitch angles without the reference plane, we introduced them to novel pitch angles (±10°, ±15°, ±20°, and ±25°). Monkeys were trained prelabyrinthectomy with a staircase procedure until they acquire ≥85% compensation and then tested with the method of constant stimuli to determine baseline behavioral performance.

### Testing schedule

Stimulus presentation was organized based on a weekly plan: on each 5-d week, our goal was to present all tested pitch angles (each day randomly interleaved 0° and two symmetrically-placed pitch angles). Animals N and P were tested 5 d/week; thus, we tested five different body tilt angles: ±10°, 15°, 20°, 25°, and 30°. Testing of animal N resumed 4 d following labyrinthectomy and included all five body pitch angles every week thereafter up to 13 weeks, with the exception of week 1 (tested for only 10°, 15°, 20°, and 25°), week 4 (tested for only 10°, 20°, 25°, and 30°), week 9 (only 10° and 30°), and week 11 (only 10°, 20°, 25°, and 30°). Animal Z was only tested 3 d/week (every other weekday), with body tilt angles of ±10°, 20°, and 30°, beginning 14 d following labyrinthectomy (with the exception of week 7 that he was only tested for 20° and 30°). During weeks 10–21, testing for animal Z resumed at five magnitudes each week (±10°, 15°, 20°, 25°, and 30°).

To confirm the efficacy of the lesions, the vestibular-ocular reflex (VOR) was measured before and after surgery with 0.5-Hz sinusoidal yaw (±10° amplitude, 31.4°/s peak velocity) and pitch (±11.2° amplitude, 35.4°/s peak velocity) rotation stimuli.

### Data analysis

We quantified behavioral performance separately for each pitch angle by fitting the proportion of “upward” choices as a function of stimulus slant in a head-centered reference frame with a cumulative Gaussian function (Psignifit toolbox for MATLAB; Wichmann and Hill, 2001a,b) to compute bias/point of subjective equality (PSE) and behavioral threshold (SD of cumulative Gaussian function), along with 95% confidence intervals. Note that, because the slant angle was increased by increment of 5°, small psychometric threshold (e.g., <5°) could not be estimated reliably. Based on each week’s data, PSE is then plotted as a function of body pitch angle and that week’s data points are fit with a linear regression. The regression slope reflects the percent compensation. If the animal performs the task perfectly, then percent compensation equals 100%.

### Sensory fusion model

We implemented a similar model as in Clemens et al. (2011) and Alberts et al. (2016). The model assumes that the brain estimates head pitch optimally based on the following information: (1) a gravity signal originating from the otoliths, encoding a pitch angle *P_OTO_* and subject to a Gaussian noise with SD *σ_OTO_*; (2) a proprioceptive gravity signal, encoding a pitch angle *P_PROP_* and subject to a Gaussian noise with SD *σ_PROP_*; and (3) a Gaussian a priori that the head is upright, centered on a pitch angle of 0°, with SD *σ_PRIOR_*.

Based on this information, the optimal estimate of pitch is the following:

(1) $$P_{FINAL}=w_{OTO}.P_{OTO}+w_{PROP}.P_{PROP}+w_{PRIOR}.0.$$

With

(2a) $$w_{OTO}=\left(1/\sigma_{OTO}^{2}\right)/(1/\sigma_{OTO}^{2}+1/\sigma_{PROP}^{2}+1/\sigma_{PROP}^{2}),$$

(2b) $$w_{PROP}=\left(1/\sigma_{PROP}^{2}\right)/(1/\sigma_{OTO}^{2}+1/\sigma_{PROP}^{2}+1/\sigma_{PRIOR}^{2}),$$

(2c) $$w_{PRIOR}=\left(1/\sigma_{PRIOR}^{2}\right)/(1/\sigma_{OTO}^{2}+1/\sigma_{PROP}^{2}+1/\sigma_{PRIOR}^{2}).$$

Note that the three weights (*w_OTO_*, *w_PROP_*, and *w_PRIOR_*) always sum to 1. Therefore, although the prior does not contribute directly to *P_FINAL_* in Equation 1 (since *w_PRIOR_* is multiplied by 0), it does so indirectly since a large prior weight necessarily reduces the vestibular and proprioceptive weights.

Furthermore, the SD of *P_FINAL_* is the following:

(3) $$\sigma_{FINAL}=sqrt(w_{OTO}^{2}.\sigma_{OTO}^{2}+w_{PROP}^{2}.\sigma_{PROP}^{2}).$$

Our sensory fusion model predicts that the final tilt estimate is proportional to the actual tilt of the animal (*P_STIMULUS_*). Specifically, in intact animals, both *P_OTO_* and *P_PROP_* are equal to *P_STIMULUS_*, leading to:

(4a) $$P_{FINAL}=(w_{OTO}+w_{PROP}).P_{STIMULUS}.$$

Following BVL, the otolith signal is set to 0, leading to:

(4b) $$P_{FINAL}=w_{PROP}.P_{STIMULUS}.$$

**Figure 2.**
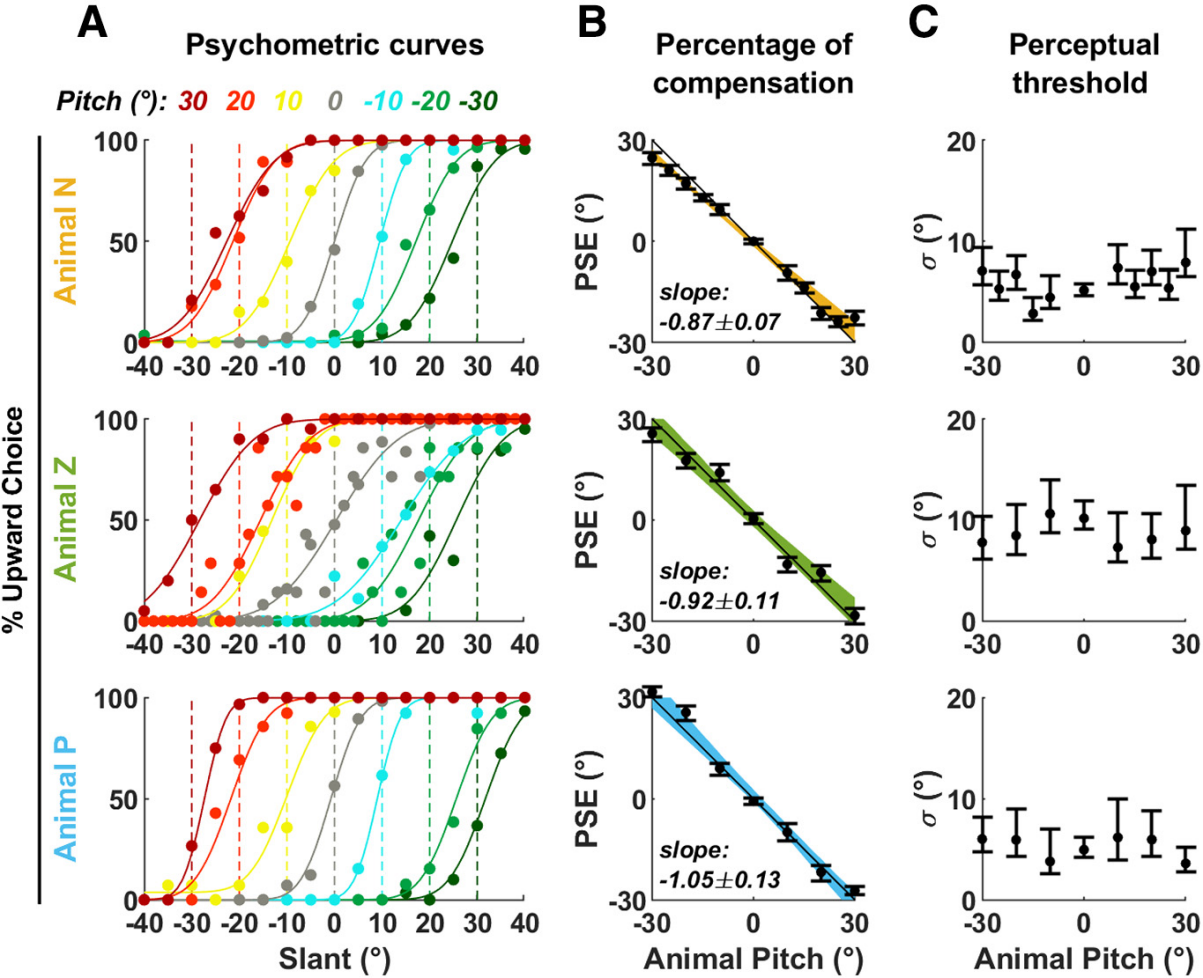
Summary of responses in intact animals. ***A***, Psychometric curves (% of upward choice as a function of visual slant), color coded based on the animal’s pitch. Dots: data points; solid lines: curve fits. The colored broken lines indicate the positions at which the curves’ PSE (i.e., 50% upward choice) should be if the animals perform the task perfectly. ***B***, PSE as a function of pitch angle. Black dots and error bars represent the mean PSE and the 95% interval of the mean. Color bands represent the 95% confidence interval of a linear regression between PSE and pitch angle. The percentage of compensation is the opposite of the slope of the regression line. The slope and 95% interval of the slope are indicated. ***C***, Perceptual threshold as a function of pitch angle, obtained from the curve fits in ***A***. Error bars represent the 95% confidence interval.

**Figure 3.**
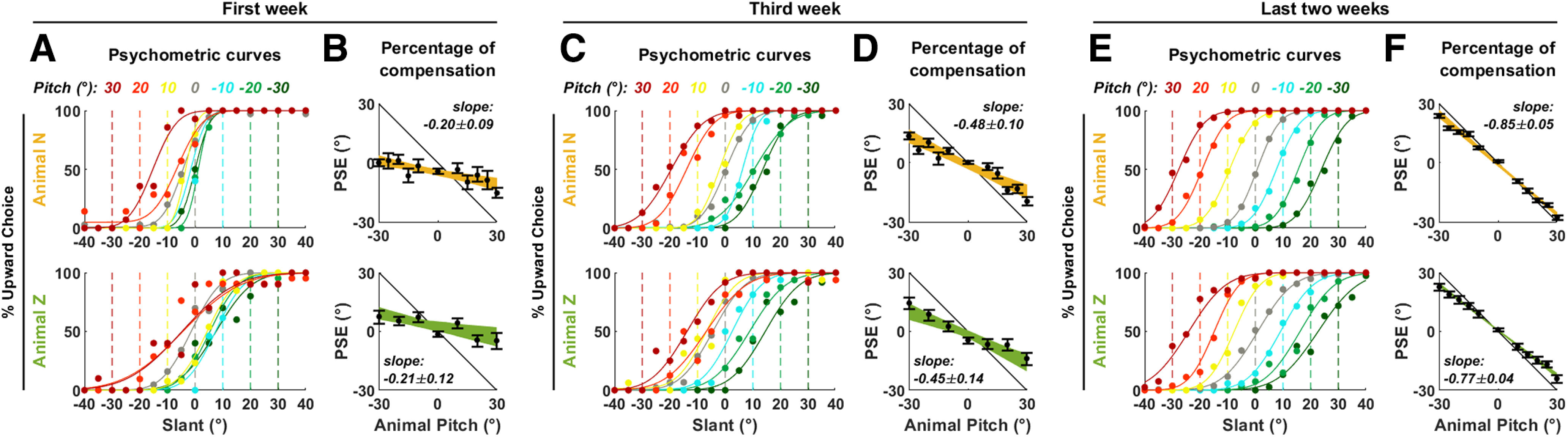
Animal performance following labyrinthectomy. Psychometric curves and percentage of compensation in animals N and Z (***A***, ***B***) immediately following labyrinthectomy, (***C***, ***D***) during the third week, and (***E***, ***F***) during last two weeks of recordings. Same legend as in Figure 2*A*,*B*.

We assume that the PSE measured during the SVV task reflects the final pitch estimate (*P_FINAL_*). As a consequence, the coefficient of proportionality (*w_OTO_* + *w_PROP_* in Eq. 4a and *w_PROP_* in Eq. 4b) corresponds to the percentage of compensation measured in Figures 2*B*, 3*B*,*D*,*F*.

We model the brain’s adaptation to BVL as follows: immediately after BVL, we assume that the brain uses the same sensory weights as in intact animals. Next, we assume that the brain adapts by recomputing optimal weights *w_PROP_* and *w_PRIOR_* in the absence of vestibular inputs. These weights can be computed by removing the term *1/σ^2^_OTO_* from Equations 2b, 2c.

We used a gradient ascent procedure to match the model’s prediction to experimental data in animals N and Z. Specifically, we adjusted the parameters to reproduce that average percentage of compensation across these animals, before (0.895) and after (0.205) labyrinthectomy and following adaptation (0.81). Furthermore, we also adjusted them such that *σ_FINAL_* matches the average psychometric threshold (3.6°) measured in Elmore et al. (2019).

Note that our model is identical as those of Clemens et al. (2011) and Alberts et al. (2016), except that these models assumed that vestibular and proprioceptive noises are tilt dependent, i.e., *σ_OTO_* and *σ_PROP_* increase as a function of *P_STIMULUS_*. This allows reproducing response non-linearities that occur when SVV is measured across a wider range of tilt (±120°), where the PSE does not vary linearly as a function of head tilt. Here, we found that PSEs were linear across a tilt range of ±30°, and therefore simply used constant noise parameters *σ_OTO_* and *σ_PROP_*, which prevent our model from being underdetermined.

### Data availability

Data supporting the study is available from the authors on reasonable request.

## Results

### Intact macaques learn to transform head-fixed visual slant in an allocentric, gravity-referenced frame

We trained three macaque monkeys to discriminate the orientation of a stereoscopically displayed visual plane relative to an allocentric earth-vertical plane, as animals, together with the visual display, were tilted in pitch (Fig. 1). Thus, the task was a SVV task adapted to rotation in the pitch plane, and required converting egocentric visual slant into an allocentric reference frame using allocentric earth-vertical cues which could only arise from vestibular and/or proprioceptive graviceptive signals.

To quantify the animal’s performance, we computed psychometric response curves as a function of egocentric visual slant (Fig. 2*A*). If animals perform the task accurately, the curves recorded at each pitch position (Fig. 2*A*, color code) should shift by an amount opposite to the animal’s pitch (Fig. 2*A*, broken vertical lines), such that the PSE, i.e., the visual angle at which a surface appears vertical, would correspond to the egocentric position of the allocentric vertical plane. In all animals, we observed that the curves shifted in an approximately compensatory manner (Fig. 2*A*). Importantly, the visual stimuli, including the range of slant angles tested, were always identical (relative to the animal’s head) and independent of the monkey’s pitch angle. Thus, the observed behavioral shifts, which show a consistent and systematic dependence on monkey pitch tilt (Fig. 2*A*), do not merely represent a response bias but a true reference frame transformation.

We computed the animal’s percentage of pitch compensation by performing a linear regression between PSE and pitch (Fig. 2*B*); a slope of −1 corresponding to 100% compensation. All animals showed a systematic compensation for pitch (animal Z: 92 ± 11%; animal P: 104 ± 14%; animal N: 86 ± 7% compensation). We did not find a dependence of perceptual threshold on the animal’s tilt (Fig. 2*C*), but the experimental protocol was not optimized to measure threshold (see Materials and Methods, Data analysis). Thus, in the following analysis, we focus our comparisons to the shifts in psychometric functions.

### Acute labyrinthectomy impairs gravity-referenced visual transformation

**Figure 4.**
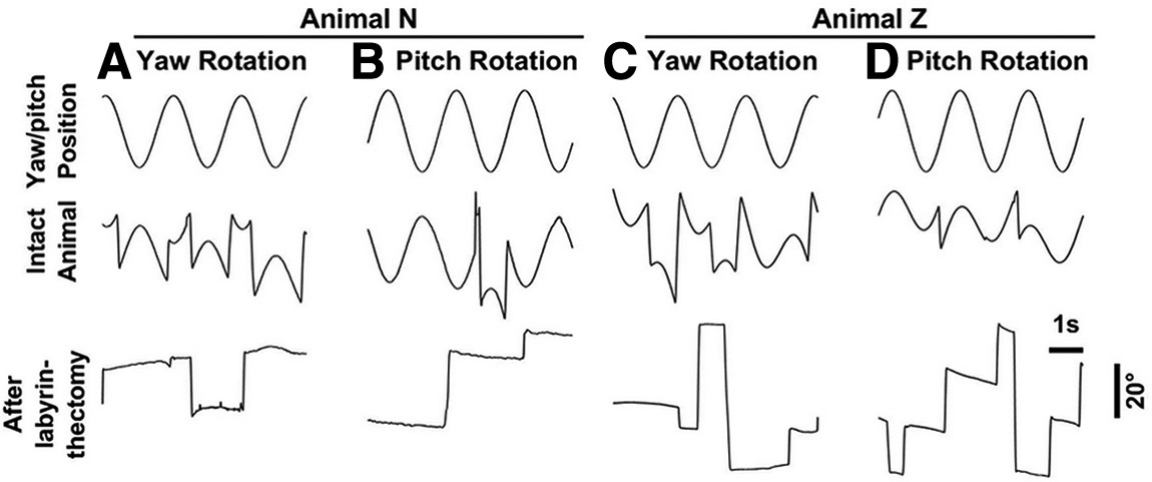
VOR. ***A–D***, Horizontal or vertical eye position during three cycles of yaw (0.5 Hz, ±10°; top) or pitch (0.5 Hz, ±11.2°) rotation in darkness, before (middle) and after (bottom).

To ascertain the role of the vestibular system in sensing gravity, we surgically destroyed the vestibular labyrinths bilaterally in two animals (N and Z) and repeated the allocentric task at days 4–11 (animal N) and 14–21 (animal Z; Fig. 3*A*,*B*). In both animals, the ability to integrate gravity information was severely compromised. Individual psychometric curves only shifted to a limited extent to compensate for the animal’s pitch (Fig. 3*A*) and, on average, the animal’s percentage of tilt compensation decreased to ∼20% (animal N: 20 ± 8%; animal Z: 21 ± 13% compensation; Fig. 3*B*). The effectiveness of labyrinthectomies was ascertained by the absence of the VOR (Fig. 4).

### The ability to perform gravity-referenced visual transformation recovers following labyrinthectomy

**Figure 5.**
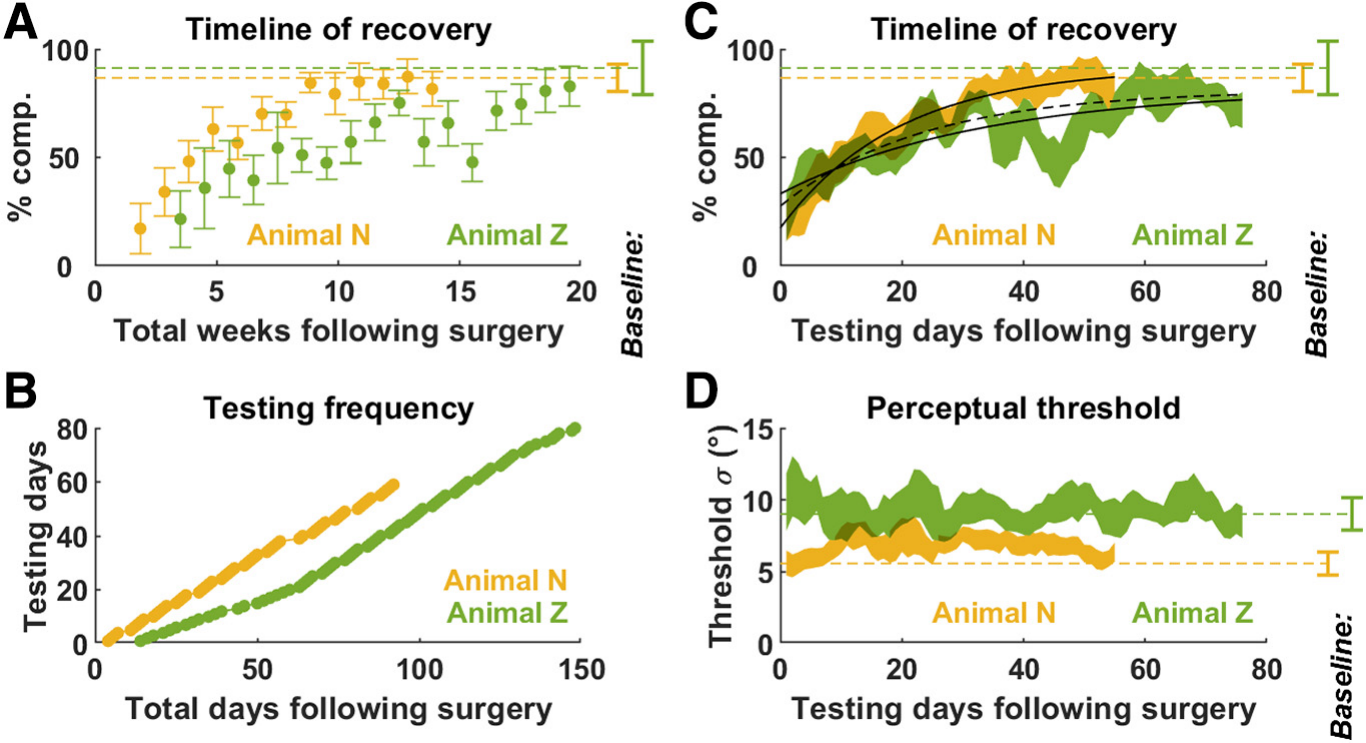
Dynamics of recovery following labyrinthectomy. ***A*,** Percentage of compensation as a function of time (number of weeks) since labyrinthectomy in animals N (yellow) and Z (green). Bars represent 95% confidence intervals. ***B***, Different testing schedule for animals N and Z after labyrinthectomy. ***C***, Same data as in panel ***A***, but displayed as a function of testing days since labyrinthectomy. Data are pooled over a 5-d time window (±2 d around each time point), and the percentage of compensation is computed as in Figure 2*B*. The colored bands represent the 95% confidence interval of the percentage of compensation. Exponential curve fits to the average percentage of compensation are shown by solid black curves; the broken black curve represents curve fitted in animal Z where data between 30 and 60 d is excluded. ***D***, Average perceptual threshold (across all psychometric curves recorded within a 5-d time window) as a function of the number of testing days since labyrinthectomy. The colored bands represent the confidence interval of the mean. In ***A***, ***C***, ***D***, the baseline performance before labyrinthectomy is represented by broken lines and confidence intervals on the right side of the plots.

Despite total loss of vestibular function, we found that the animals’ performance gradually recovered over a period of weeks (Figs. 3*C****–****F*, 5*A*). We estimated the percentage of compensation as a function of the number of experimental testing/training days following surgery (which differed from the total days after surgery; Fig. 5*B*). We found that both animals recovered at a remarkably similar rate, mostly during the first few weeks, where the two curves were practically superimposed (Fig. 5*C*). On the third week of testing (animal N: 18–22 d; animal Z: 28–32 d following surgery), the percentage of compensation reached 48 ± 10% (animal N) and 44 ± 13% (animal P; Fig. 3*C*,*D*). Animal N (yellow) eventually reached a performance similar to baseline (Fig. 5*C*, error bar on the right side). The performance in animal Z (green) fluctuated somewhat between *t* = 30 and 60 d before reaching stable levels after 60 d. (Fig. 3*E*,*F*).

We fitted the recovery curves with exponential functions to estimate the time constant of the recovery as well as its asymptotic performance. In animal N, gravity compensation recovered with a time constant of 19.5 d to a fitted asymptotic value of 92%, which fell in the confidence interval of baseline value (Fig. 5*C*). In animal Z, exponential fitting was affected by the fluctuation of the animal’s performance. When all experimental data were fitted, we estimated that the time constant of recovery was 33.4 d, and its asymptote 82%, within the animal’s baseline performance. When excluding data points between 30 and 60 d (Fig. 5*C*, broken black line), the asymptotic performance was similar (81%), but the estimated time constant of recovery was shorter: 23.2 d; a value that resembled that of animal N and may be more representative of the animal’s initial recovery.

### Sensory re-weighting model

**Figure 6.**
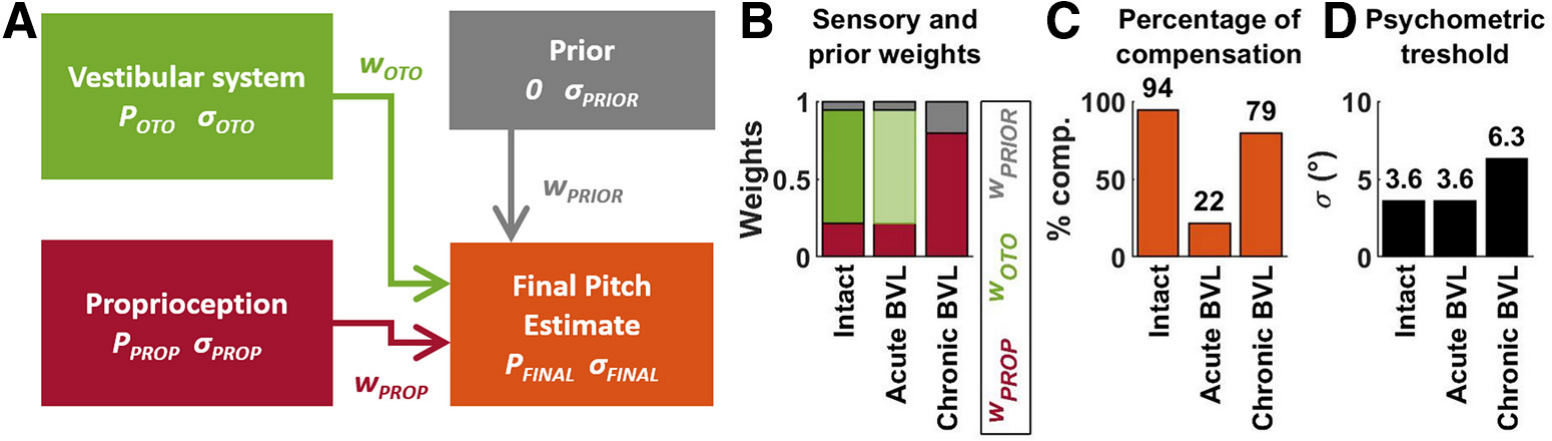
Sensory fusion model. ***A***, Overview of the model. Vestibular (green) and proprioceptive (red) sensory signals encoding head pitch, as well as prior information (gray) are combined to generate a final pitch estimate *P_FINAL_* (orange). ***B–D***, Model simulation. Panel ***B*** shows the optimal sensory weights in intact animals and following BVL. Same color code as in ***A***. After BVL, the vestibular weight is shown in light green color to emphasize that the vestibular modality is silenced although its weight is unchanged. Panel ***C*** shows the total weight of sensory signals, which corresponds to the percentage of compensation. Panel ***D*** shows the variability of the final pitch estimate.

In order to compare our results with previous experimental and modeling studies, we adapted the sensory fusion models of Clemens et al. (2011) and Alberts et al. (2016; Fig. 6*A*). We assumed that pitch signals originating from the otoliths and proprioception (*P_OTO_* and *P_PROP_*) are weighted (weights *w_OTO_* and *w_PROP_*) to form a final pitch estimate. The brain also uses information about the a priori statistical distribution of head pitch, which is modeled as a Gaussian centered on 0 with SD *σ_PRIOR_*. The weights of the sensory modalities (*w_OTO_* and *w_PROP_*) and of the prior (*w_PRIOR_*) are determined by the amount of sensory noise (*σ_OTO_* and *σ_PROP_*) as well as the SD of the prior (*σ_PRIOR_*), more accurate (i.e., lower *σ*) modalities being more heavily weighted (see Materials and Methods). Note that these weights always sum up to 1 (Fig. 6*B*); therefore, the prior weight *w_PRIOR_* reduces the other sensory weights and biases the final estimate toward 0. During SVV, the percentage of compensation is equal to the total sensory weight (*w_OTO_*+ *w_PROP_* in intact animals, *w_PROP_* following BVL).

We fitted the model’s free parameters (*σ_OTO_*, *σ_PROP_*, and *σ_PRIOR_*) to reproduce the percentage of compensation in animals N and Z, as well as the average psychometric threshold, measured in Elmore et al. (2019; 3.6°) and analyzed the prediction of the best-fitting model (*σ_OTO_* = 4.3°, *σ_PROP_* = 7.9°, and *σ_PRIOR_* = 15.6°). In intact animals, the vestibular signal’s weight is ∼3.5 times larger compare to the proprioceptive weight (Fig. 6*B*, 0.73 vs 0.21). The sum of these weights (0.94; Fig. 6*C*) corresponds to the percentage of compensation in intact animals (Fig. 2*B*, 0.89 in animals N and Z). In contrast, after BVL, the vestibular modality disappears. We assumed that, in an acute stage, the sensory weights are identical as in intact animals. In this situation, the remaining proprioceptive weight of 0.22 matches the percentage of compensation measured immediately after labyrinthectomy (Fig. 3*B*). We finally modeled chronic BVL by re-computing the optimal weights once the vestibular modality is removed from the model. We found that proprioceptive weight (and the corresponding percentage of compensation) increases to 0.79 (Fig. 6*B*,*C*), in agreement with our experimental findings (Fig. 3*F*). The model also predicts (Fig. 6*D*) that loss of vestibular function should lead to an increase of the psychometric threshold from 3.6° (consistent with the value measured Elmore et al., 2019) to 6.4° (which, however, could not be measured accurately in this study). These results indicate that the fundamental assumptions that underlie most current models of verticality sensing are sufficient to explain the acute and chronic effects of BVL.

## Discussion

We have measured the relative contributions of vestibular and proprioceptive cues in the estimation of gravity used for visual orientation perception in a SVV task adapted to the pitch plane. Macaques estimated the visual slant with respect to earth-vertical at different body pitch orientations. The animals and the visual display were tilted together relative the allocentric vertical, such that completing the task required combining egocentric visual slant with allocentric body tilt, which could only be sensed through graviception. We found that bilateral labyrinthectomy resulted in a dramatic decrease in the animal’s ability to sense body tilt, dropping from 90% to 20%, that should represent the contribution of proprioception in labyrinth-intact animals (although this value may be slightly overestimated since animals were tested a few days following surgery). We also deduce that the contribution of otolithic graviception in intact animals should be about ∼70%. We found that both animals improved over several weeks, reaching performance similar to baseline, with a time constant of approximately three weeks of testing/retraining. This indicates that extravestibular gravity cues, which may originate from body graviception, gradually compensate for vestibular loss. The rate of recovery was similar across animals and, remarkably, seemed to be governed by the number of experimental sessions, rather than the absolute time elapsed since the surgery. This indicates that compensation is promoted by exercising the task. Note that one may have expected that the animals start recovering as soon as they start locomoting in their cages. Therefore, it is possible that recovery may be task specific.

Our findings parallel the time course of clinical symptoms following vestibular loss, that usually induces severe balance and spatial orientation deficits that recover over time (Crawford, 1964; Gillespie and Minor, 1999; Brown et al., 2001; Zingler et al., 2008; Ward et al., 2013; Strupp et al., 2016). Other studies (Tabak et al., 1997; Lopez et al., 2007; Grabherr et al., 2011; Alberts et al., 2015, 2018; Toupet et al., 2017; Bürgin et al., 2018) found that SVV was approximately normal, although more variable (Bürgin et al., 2018), in human patients, although none of these studies captured the time course of recovery. Note that the SVV task probes graviception specifically. Other vestibular functions, such as rotation perception and gaze stabilization, are permanently affected following BVL and contribute to clinical outcomes (Crane and Demer, 1998; Guinand et al., 2012; Hain et al., 2013; Petersen et al., 2013).

Optimal sensory fusion posits that multiple sources of information are combined according to their respective reliability. In healthy subjects, both vestibular and proprioception should therefore contribute to graviception, although the former should have a greater weight. Our study provides, for the first time, a direct measure of these weights. Indeed, the percentage of compensation measured immediately after labyrinthectomy should reflect the proprioceptive weight. Furthermore, the difference between the percentage of compensation measured before and immediately after labyrinthectomy should reflect the vestibular weight. Here, we found that the proprioceptive weight was ∼20% in both animals, and the vestibular weight ∼70%. Optimal sensory fusion also predicts that the brain should reweight proprioceptive signals following labyrinthectomy, in agreement with our findings. It is notable that the best-fitting parameters (i.e., the SDs of vestibular and proprioceptive sensory noise, as well as the prior distribution of head tilt; *σ_OTO_*, *σ_PROP_*, and *σ_PRIOR_*, respectively) in our study resemble those found in Clemens et al. (2011) and Alberts et al. (2016). These two models assumed that sensory noise were tilt dependent; however, when average in the range of ±30° used here, we find Clemens et al. (2011) and Alberts et al. (2016) predict a vestibular noise of 5.1° and 7.8°, respectively (vs 4.3° in our study); a proprioceptive noise of a 11.9° and 7.5°, respectively (vs 7.9° in our study), and a prior of 12.5° in both models (vs 15.6° in our study). This indicates that the optimal sensory fusion model generalizes well over species. Optimal estimation also predicts that, if other sensory cues are provided, their weight should increase following BVL. Accordingly, other studies (Lopez et al., 2007; Toupet et al., 2017; Alberts et al., 2018) found that visual cues about allocentric tilt had a greater impact on SVV following vestibular loss. Thus, optimal sensory fusion has proven to be a largely successful framework for studying verticality perception in healthy subjects (De Vrijer et al., 2008, 2009; Tarnutzer et al., 2009a,b; Clemens et al., 2011; Alberts et al., 2016) or following vestibular deficits (Alberts et al., 2015, 2018). More generally, optimal estimation models (Oman, 1982; Borah et al., 1988; Merfeld, 1995; Glasauer and Merfeld, 1997; Laurens and Droulez, 2007; MacNeilage et al., 2007; Laurens and Angelaki, 2011, 2017; Karmali and Merfeld, 2012) are now the predominant approach to model vestibular information processing.

Comparing our study to previous work in humans indicates that verticality perception follows similar principles in humans and macaque monkeys with a rather close degree of quantitative agreement. This similarity is also supported by previous SVV studies in macaques (Daddaoua et al., 2008; Lewis et al., 2008). In particular, Lewis et al. (2008) demonstrated that the dynamics of illusory tilt experienced when rotated in a centrifuge is similar as in humans (Clark and Graybiel, 1966), an observation that strongly supports optimal model theories (Merfeld, 1995; Bos and Bles, 2002; Laurens and Droulez, 2007).

SVV tasks represent a clinical test of otolith function, commonly used to detect otolith imbalance that results from and persists following unilateral vestibular loss (Böhmer and Rickenmann, 1995; Valko et al., 2011) or central lesions (Halmagyi et al., 1990; Brandt et al., 1994; Dieterich and Brandt, 2019). In contrast, our study (see also Tabak et al., 1997; Lopez et al., 2007; Grabherr et al., 2011; Alberts et al., 2015, 2018; Toupet et al., 2017; Bürgin et al., 2018) demonstrates that sensory compensation is sufficient to allow near to normal SVV performance following vestibular loss, except for a moderate increase in response variability (Bürgin et al., 2018). This indicates that assessing otolith function should generally be performed using other techniques, such as posturography (Brown et al., 2001) or ocular vestibular evoked potentials (Valko et al., 2011; Bürgin et al., 2018).

The majority of SVV studies (Daddaoua et al., 2008; De Vrijer et al., 2008, 2009; Kaptein and Van Gisbergen, 2006; Lewis et al., 2008; Clemens et al., 2011; Alberts et al., 2015, 2016, 2018; Bürgin et al., 2018) have been performed by tilting subjects in the roll plane. These studies generally report a typical pattern of error where subjects overestimate small tilt angles (e.g., <30°; “E-effect”) and underestimate large tilt angles (e.g., 60–150°, “A-effect”). One study (Ebenholtz, 1970) measured SVV in humans during both pitch and roll tilt, and found similar patterns of overestimation and underestimation. Here, we designed a SVV task where animals reported the allocentric orientation of a visual plane while being tilted in the pitch plane. We used a restricted range of body tilt, in which the A-effect does not occur. Furthermore, we did not observe any E-effect. This likely happened because the E-effect during head tilt is linked to a reflexive eye counterrotation that stabilized the eye in space. In our task, we required animals to fixate a head-centered point, effectively suppressing the ocular counterrotation and likely the E-effect. Therefore, our results in intact animals, in which pitch angles up to 30° were sensed accurately, are consistent with previous SVV studies. Furthermore, our modeling results resemble those of previous studies that measured SVV in the roll plane in humans (Clemens et al., 2011; Alberts et al., 2016). This indicates that our results are likely representative of SVV tasks performed at small tilt angles (<30°), in the pitch and roll planes, in humans and macaque monkeys.

Note that, rather than bars, here we used planar surfaces because they elicit robust responses from higher visual areas that may play a key role in creating a gravity-centered visual representation (Rosenberg et al., 2013; Rosenberg and Angelaki, 2014). Further, rather than using a manual bar task in which subjects physically align a bar with the earth-vertical (as in previous studies; see Daddaoua et al., 2008; De Vrijer et al., 2008; Kaptein and Van Gisbergen, 2006; Lewis et al., 2008; Bürgin et al., 2018), we have used a two-alternative-forced-choice slant discrimination task, as more recent human studies have employed (De Vrijer et al., 2009; Clemens et al., 2011; Alberts et al., 2015, 2016, 2018). Finally, although SVV studies typically consider roll tilts, here, we have generalized the findings to pitch orientations. Perception of visual slant orientation during pitch body tilts involves computation of orientation in depth, whose neural mechanisms are likely more intensive computationally.

How does the brain transform egocentrically encoded visual information into the gravity-centered representation of the world we perceive? A few older studies suggested early visual cortex may be involved (Denney and Adorjani, 1972; Horn et al., 1972; Sauvan, 1999; Tomko et al., 1981) but were subsequently refuted (Daddaoua et al., 2014). It is now well accepted that visual signals are first encoded in retinal coordinates. Thus, somewhere in the neural representation of the three-dimensional visual scene, multisensory gravity cues must interact with visual orientation signals to generate an allocentric representation. Where and how a gravity-centered, allocentric representation is achieved in the brain is unknown. Visual adaptation experiments (Mitchell and Blakemore, 1972) and clinical studies (Brandt et al., 1994; Funk et al., 2010, 2011; Guardia et al., 2012; Rousseaux et al., 2015) suggest that a gravity-centered representation of object orientation arises in higher cortical areas. One candidate area is the central intraparietal area, where many neurons encoded visual plane orientation in a gravity-centered or intermediate reference frame (Rosenberg and Angelaki, 2014). Another area, suggested by clinical work, is the human homolog of parieto-insular vestibular cortex (Brandt et al., 1994; Baier et. al, 2012b; Funk et al., 2010, 2011) or temporo-parietal cortex (Kheradmand and Winnick, 2017). This broad area in human cortex additionally includes the homolog of the macaque visual posterior sylvian area, also known as “parieto-temporal association area T3” (Jones and Burton, 1976; Grüsser et al., 1990a,b; Guldin et al., 1992; Guldin and Grüsser, 1998; Dicke et al., 2008). Finally, brainstem and thalamic regions have also been implicated (Baier et al., 2012a, 2016, 2017). It is important that future studies explore the neural circuits mediating SVV perception.

## Synthesis

Reviewing Editor: Leonard Maler, University of Ottawa Decisions are customarily a result of the Reviewing Editor and the peer reviewers coming together and discussing their recommendations until a consensus is reached. When revisions are invited, a fact-based synthesis statement explaining their decision and outlining what is needed to prepare a revision will be listed below. The following reviewer(s) agreed to reveal their identity: Alexander Tarnutzer, Jerome Carriot.

Editorial comments

Both reviewers and the Editor agree that this is an important paper that reaches high scientific standard and should be published.

Both reviewers have found a number of relatively minor issues that need to be addressed. Their reviews are thorough and complete and so I have presented them nearly verbatim below. Please be sure to address each point in a rebuttal and also provide a redlined copy; this will enable a fast re-review of an excellent contribution.

Reviewer #1

Overall, the work presented here is novel and provides new and important insights into mechanisms of recovery after bilateral vestibular loss. Due to the complexity of training monkeys for such a behavioral task as indicating perceived direction of gravity, few studies have addressed this topic (see also Lewis et al. J Neurophysiol. 2008 Jul;100(1):140-53. doi: 10.1152/jn.01012.2007) and Daddaoua et al. J Vis. 2008 Mar 24;8(3):19.1-8. doi: 10.1167/8.3.19). While I do not see any major issues regarding this study, several points should be considered by the authors as enlisted below. This includes better distinguishing human and non-human studies related to the topic in the introduction and XXX

Specific comments:

Abstract:

Line 7: please state that vestibular loss is bilateral

Line 7-8: please state how graviceptive gain is defined.

Introduction:

General comment: when summarizing previous work done on the field it remains unclear which studies have been performed in humans and which ones in non-human primates (Daddaoua et al. 2008, Lewis et al. 2008). Considering that the work presented here focuses on verticality perception in non-human primates a more detailed discussion of previous work in non-human primates should be provided.

General comment II: in the introduction paradigms (and published studies) applied in the roll plane are presented. Thus, the authors need to explain why they opted for studying verticality perception in the pitch plane.

Line 63-4: I suggest adding the following citation: Schuler et al. Clinical Neurophysiology 129 (2018) 934-945

Line 63-4: please specify that this relates to SVV accuracy, and not SVV precision (which is reduced...see Schuler et al. 2018)

Materials and methods

Line 84 and following: Did the animals receive any antivertiginous medication post surgery? This should be stated and if yes, it should also be stated for how many days such medication was applied. This is important as antiemetics have a negative impact on central compensation after peripheral-vestibular loss.

Results:

Lines 207-216: These paragraphs rather belong to the methods section.

Discussion:

Lines 310-13: I suggest further distinguishing between SVV accuracy and SVV precision. See also the additional citation (Schuler et al. Clinical Neurophysiology 129 (2018) 934-945)

Lines 313-5: The authors may add citations here to underline this statement.

Lines 343-6: This should be clarified, as in the current study the focus was on SVV accuracy, whereas no results on SVV precision (i.e., trial-to-trial variability) were presented. From human patients it is known that the precision of SVV estimates in bilateral vestibular loss is reduced (See e.g. Schuler et al. 2018).

Lines 346-8: calculating SVV precision is also an alternative approach to assess otolith function in these patients (see Schuler et al. 2018).

Reviewer #2

This study present data on a spatial orientation psychophysic task in NHP. It demonstrates that perception of body orientation mainly rely on the vestibular system. However, in case of vestibular loss cross modal plasticity occurs and graviception is recovered to nearly pre-lesion levels in about 3 weeks.

I am enthusiastic with this study. It will probably be the go-to study to future manuscripts about neuronal mechanisms underlying the perception of spatial orientation. This is a long-awaited data set that I am glad to see published. Nevertheless, in my opinion, because of its potential, few issues should be addressed. My comments boil down to three topics: 1) the dimension the animal is tilted; 2) the psychophysic task used to assess graviception and 3) the recovery period.

1) Most of the studies that have addressed the SVV have done it during roll tilt. To my knowledge only Ebenholtz 1970 used the SVV in pitch. Other studies have used different psychophysic tasks (Subjective vertical, horizon etc...) and found differences between roll and pitch (only a Muller effect in pitch and Muller and Aubert effects in roll). This difference is unusually explained by unbalanced touch and pressure cues between pitch forward vs backward, whereas touch and pressure cues are balanced between left hear down and right hear down roll tilt.

As a speculation, if Clemens et al., 2011 had used pitch they probably have seen a different proprioceptive weight between pitch forward and backward. On another note, how long was the monkey held in position after being tilted? Do you need to consider the canal time constant in the model?

2) I have trouble considering the present task as a SVV task. The SVV has been described for the last ∼150 years as a strike of light perceived as parallel to the gravity in the fronto-parallel plan. However, I agree that the presented task is a “graviceptive/spatial orientation perception task", if the monkeys actually choose the target that passes the vertical plan. This is where I have some concerns. If I understood correctly Elmore et al., 2019, the same monkeys that were used in the present study were first trained to make a saccade to the upper target if the plan was slanted away (positive direction in the study) from them. Would it be possible that it continues to perform the same task? During training in the tilted condition, the vertical plan was flashed. Would it be possible that the monkey simply changed its reference? Is there a simple way to show that the monkeys actually perform a graviceptive task and not an adapted version of the former task they were trained for?

3) I am puzzled that both monkeys recover at a similar rate relative to the experimental procedure. As soon as the monkey starts moving in its cage after the surgery, sensory substitution should begin and Animal Z should have a higher proprioceptive weight at the beginning of the testing. As it stands, it looks like the recovery is specific to the task.

details: -line 323: the word “about” is repeated twice.

- I am not convince that"proprioception accounts for up to 20% of gravity sensing” is the highlight of the paper. There is so much more.

## Author Response

Answer to reviewers

Reviewer #1

Overall, the work presented here is novel and provides new and important insights into mechanisms of recovery after bilateral vestibular loss. Due to the complexity of training monkeys for such a behavioral task as indicating perceived direction of gravity, few studies have addressed this topic (see also Lewis et al. J Neurophysiol. 2008 Jul;100(1):140-53. doi: 10.1152/jn.01012.2007) and Daddaoua et al. J Vis. 2008 Mar 24;8(3):19.1-8. doi: 10.1167/8.3.19). While I do not see any major issues regarding this study, several points should be considered by the authors as enlisted below. This includes better distinguishing human and non-human studies related to the topic in the introduction.

We thank the reviewer for these supportive comments.

Specific comments:

Abstract:

Line 7: please state that vestibular loss is bilateral

Done

Line 7-8: please state how graviceptive gain is defined.

Done

Introduction:

General comment: when summarizing previous work done on the field it remains unclear which studies have been performed in humans and which ones in non-human primates (Daddaoua et al. 2008, Lewis et al. 2008). Considering that the work presented here focuses on verticality perception in non-human primates a more detailed discussion of previous work in non-human primates should be provided.

We now introduce studies in human and macaques separately; and we added a paragraph to the discussion to discuss the similarities between verticality perception in humans and macaques.

General comment II: in the introduction paradigms (and published studies) applied in the roll plane are presented. Thus, the authors need to explain why they opted for studying verticality perception in the pitch plane.

We opted for studying verticality perception in the pitch plane because we had the opportunity to use animals that were already trained at a slant discrimination task. Beyond practical considerations, using these animals allowed us to adhere to the ethical principle of reducing the number of animals used in our laboratory.

We added a paragraph to the discussion to discuss the similarities between SVV tasks in the roll and pitch planes.

Line 63-4: I suggest adding the following citation: Schuler et al. Clinical Neurophysiology 129 (2018) 934-945

Line 63-4: please specify that this relates to SVV accuracy, and not SVV precision (which is reduced...see Schuler et al. 2018)

Lines 310-13: I suggest further distinguishing between SVV accuracy and SVV precision. See also the additional citation (Schuler et al. Clinical Neurophysiology 129 (2018) 934-945)

Lines 343-6: This should be clarified, as in the current study the focus was on SVV accuracy, whereas no results on SVV precision (i.e., trial-to-trial variability) were presented. From human patients it is known that the precision of SVV estimates in bilateral vestibular loss is reduced (See e.g. Schuler et al. 2018).

Lines 346-8: calculating SVV precision is also an alternative approach to assess otolith function in these patients (see Schuler et al. 2018).

We thank the reviewer for bringing this citation (it is in fact by A. Bürgin et al.) to our attention. We have grouped here all comments referring to this study, and we discuss briefly how this study relates to our findings. Bürgin et al. found that the A-effect was essentially similar between healthy control and BVL patients, but that SVV variability increased (i.e. precision decreased) in patients. This matches relatively well results by Alberts et al. (2015) who found also that the A-effect was the same (this study didn’t observe a change in variability; however this may be due to the low number of patients).

In our study, we measured psychometric curves by presenting visual slant angles at 5{degree sign} intervals. This limited our ability to assess psychophysical thresholds, and therefore we can’t show results on precision. However, our model predicts that precision should decrease (Fig. 5D). We have mentioned the decreased in SVV precision in the discussion, as suggested.

Whether SVV precision could be used as an alternative to assess otolith functions in unclear to us: the decrease in precision is moderate, and Alberts et al. 2015 were unable to observe it. Also, Bürgin didn’t mention it as a potential diagnosis tool in his article. We feel that discussing this possibility further would raise technical questions about measuring SVV variability in a clinical setting, which may be beyond the scope of this study.

Materials and methods

Line 84 and following: Did the animals receive any antivertiginous medication post surgery? This should be stated and if yes, it should also be stated for how many days such medication was applied. This is important as antiemetics have a negative impact on central compensation after peripheral-vestibular loss.

Animals did not receive any antivertiginous medication post-surgery.

Results:

Lines 207-216: These paragraphs rather belong to the methods section.

We agree; and we shortened the first and deleted the second paragraph. We left two sentences of the first paragraph, so as to begin the results with a short description of the experiment’s rationale.

Discussion:

Lines 313-5: The authors may add citations here to underline this statement.

We added citations, as suggested.

Reviewer #2

This study present data on a spatial orientation psychophysic task in NHP. It demonstrates that perception of body orientation mainly rely on the vestibular system. However, in case of vestibular loss cross modal plasticity occurs and graviception is recovered to nearly pre-lesion levels in about 3 weeks.

I am enthusiastic with this study. It will probably be the go-to study to future manuscripts about neuronal mechanisms underlying the perception of spatial orientation. This is a long-awaited data set that I am glad to see published. Nevertheless, in my opinion, because of its potential, few issues should be addressed. My comments boil down to three topics: 1) the dimension the animal is tilted; 2) the psychophysic task used to assess graviception and 3) the recovery period.

We thank the reviewer for these supportive comments.

1) Most of the studies that have addressed the SVV have done it during roll tilt. To my knowledge only Ebenholtz 1970 used the SVV in pitch. Other studies have used different psychophysic tasks (Subjective vertical, horizon etc...) and found differences between roll and pitch (only a Muller effect in pitch and Muller and Aubert effects in roll). This difference is unusually explained by unbalanced touch and pressure cues between pitch forward vs backward, whereas touch and pressure cues are balanced between left hear down and right hear down roll tilt. As a speculation, if Clemens et al., 2011 had used pitch they probably have seen a different proprioceptive weight between pitch forward and backward.

We agree that there may be quantitative differences between roll and pitch tasks in general.

Regarding the A-effect, all current modelling work model it by assuming that sensory noise of the otolith and/or proprioceptors increase at large tilt angles. Indeed, this has never been clearly demonstrated or modelled in the pitch plane. However, our study was restricted to low tilt angles where the A-effect doesn’t occur yet. Furthermore, when we compared our results to Clemens et al or later models developed in the roll plane, we found a good agreement within this range. Therefore, we would argue that our task is still representative of verticality perception in both the pitch and roll plane, in this range.

Regarding the E-effect, it is generally explained by assuming that the brain performs compensatory eye movements, but doesn’t account for these eye movements accurately. In this respect, it is distinct from the main focus of the study, which was the fusion of otolith and proprioceptive cues. It should be noted that our task may have eliminated it, since we imposed a head-fixed fixation points that eliminated compensatory eye movement in the first place.

We added one paragraph to the discussion to compare tasks in the pitch and roll plane.

On another note, how long was the monkey held in position after being tilted? Do you need to consider the canal time constant in the model?

The animals were held statically during a series of 17 trials. Therefore, the tilt movement was essentially low-frequency, such that the contribution of the semi-circular canals could be considered negligible. We pointed this out in the methods.

2) I have trouble considering the present task as a SVV task. The SVV has been described for the last ∼150 years as a strike of light perceived as parallel to the gravity in the fronto-parallel plan. However, I agree that the presented task is a “graviceptive/spatial orientation perception task", if the monkeys actually choose the target that passes the vertical plan.

We understand the reviewer’s concern. Even though we actually never referred to our task as a SVV task, we compare our task to previous SVV studies throughout the manuscript in a way may implies that they are the same.

Yet, it should be noted that our task consists in reporting the subjective orientation of a visual stimulus relative to the allocentric vertical. In this respect, it is delicate *not* to call it a SVV task. Therefore, we felt that the best way to describe it as a ‘SVV task adapted to rotation in the pitch plane’. We now do this through the manuscript.

This is where I have some concerns. If I understood correctly Elmore et al., 2019, the same monkeys that were used in the present study were first trained to make a saccade to the upper target if the plan was slanted away (positive direction in the study) from them. Would it be possible that it continues to perform the same task? During training in the tilted condition, the vertical plan was flashed. Would it be possible that the monkey simply changed its reference? Is there a simple way to show that the monkeys actually perform a graviceptive task and not an adapted version of the former task they were trained for?

Monkeys were indeed first trained as described in Elmore et al. 2019; however, this doesn’t undermine the present study. In the initial training stage, the animals were always upright, such that the egocentric and allocentric reference frames were identical. In a second stage, we started tilting the animals and trained them to report the allocentric orientation of the visual stimulus relative to an earth-vertical plane (to do so, we actually presented a visual earth-vertical plane so as to help them understand the task; this plane was gradually removed as the animals progressed).

Thus, the animals were initially trained to perform a simple task, after which the task was rendered more complex: this is the standard training procedure in Macaques. Furthermore, it is true that the second task is an “adapted” version of the initial task that requires a reference frame transformation; however this transformation can’t physically be accomplished without graviception (since it requires knowledge of the allocentric vertical, and all other sensory cues that may indicate it were withheld). Therefore, the fact that the animals could perform the task accurately (before labyrinthectomy) is sufficient to demonstrate that they were actually performing a graviceptive task.

3) I am puzzled that both monkeys recover at a similar rate relative to the experimental procedure. As soon as the monkey starts moving in its cage after the surgery, sensory substitution should begin and Animal Z should have a higher proprioceptive weight at the beginning of the testing. As it stands, it looks like the recovery is specific to the task.

This is a good point, and we mentioned it in the discussion.

details: -line 323: the word “about” is repeated twice.

Corrected.

- I am not convince that"proprioception accounts for up to 20% of gravity sensing” is the highlight of the paper. There is so much more.

We rewrote the significance statement to highlight our conclusions better.
